# Ipilimumab-Induced Neutropenia in Melanoma

**DOI:** 10.1177/2324709616661835

**Published:** 2016-08-09

**Authors:** Makiko Ban-Hoefen, Richard Burack, Lynn Sievert, Deepak Sahasrabudhe

**Affiliations:** 1 University of Rochester Medical Center, Rochester, NY, USA

**Keywords:** checkpoint inhibitors, ipilimumab, immune-mediated, neutropenia, melanoma

## Abstract

Ipilimumab is a human monoclonal IgG1 antibody against CTLA-4 that has been shown to prolong the overall survival of advanced melanoma. The most common adverse events associated with ipilimumab are immune-related. Severe hematological toxicity is rare. We report a case of severe neutropenia following ipilimumab therapy that fully resolved after the administration of prednisone, cyclosporine, and anti-thymocyte globulin therapies.

## Case Presentation

We present the case of a 54-year-old man with a T4b N1 M0 (stage IIIB) cutaneous melanoma located on the dorsum of his right foot. He underwent wide excision of the lesion as well as a sentinel lymph node biopsy and a right inguinal lymph node dissection. He enrolled on the intergroup clinical trial E1609, a phase III randomized study of adjuvant ipilimumab versus high-dose interferon-α2B for resected high-risk melanoma. He was assigned to receive ipilimumab 10 mg/kg intravenously (IV) every 3 weeks for 4 doses and then every 3 months for 3 doses thereafter as adjuvant therapy.

**Figure 1.**
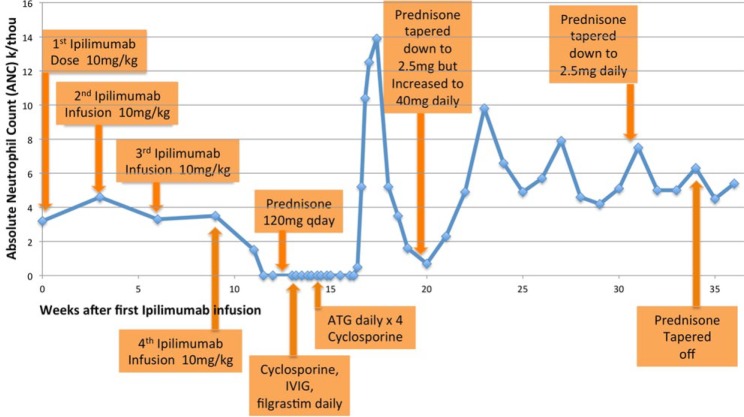
Absolute neutrophil count (ANC) in weeks after first ipilimumab infusion.

The patient’s clinical course is detailed in Table 1 and depicted in graph format in Figure 1. He received 4 doses of ipilimumab at 10 mg/kg IV every 3 weeks, with the last dose given 9 weeks after initiation. His complete blood count and comprehensive metabolic panel were monitored weekly. He developed a maculopapular rash on his torso and arms within 1 week of the first infusion. The rash improved with the application of 2.5% hydrocortisone cream. He also experienced decreased libido. Due to concerns for panhypopituitarism as a side effect of ipilimumab, luteinizing hormone, follicle-stimulating hormone, and testosterone levels were checked. While luteinizing hormone and follicle-stimulating hormone levels were in the normal range (6·5 miU/mL and 4·8 miU/mL, respectively), the testosterone level was mildly low at 145 ng/dL. Therefore, topical testosterone 5 g topical daily was prescribed with improvement in his libido. Approximately 2 weeks after the fourth dose of ipilimumab, the patient developed a sore throat, fevers (to a maximum of 38.2°C), dyspnea, and worsening fatigue. His absolute neutrophil count (ANC) was 0·0 × 10^9^/L, having been last documented normal 2 weeks prior to the fourth dose of ipilimumab.

**Table 1.** Clinical Course^a^.

Week	Treatment/Intervention	Patient’s Symptoms and Clinical Findings	WBC (× 10^9^/L)	ANC (× 10^9^/L)	Hct (%)	Platelet (× 10^9^/L)
0	First infusion of ipilimumab (10 mg/kg)	Rash on torso and arms a week after infusion	5.6	3.2	42	263
3	Second infusion of ipilimumab (10 mg/kg)		8.2	4.6	45	237
6	Third infusion of ipilimumab (10 mg/kg)		6.7	3.3	45	261
9	Fourth infusion of ipilimumab (10 mg/kg)	Rash improved w/2.5% hydrocortisone cream	8.3	3.5	47	225
11			6.4	**1.5**	44	183
		Sore throat, fevers, dyspnea, and fatigue	5.1	**0.0**	41	173
12		UTI with enterococcus; bone marrow biopsy	**4.0**	**0.0**	40	221
13	Started prednisone 60 mg PO BID	Neutropenic fever	**2.8**	**0.0**	**38**	238
	Cyclosoprine 125 mg PO BID × 5 days; IVIG 40 g IV daily × 4 doses; and filgrastim 5 µg/kg daily × 5 doses started		**2.1**	**0.0**	**34**	305
14	Prednisone decreased to 50 mg BID	Perirectal pain	**0.7**	**0.0**	**33**	223
	Prednisone deceased to 40 mg BID		**0.9**	**0.0**	**38**	203
	ATG 15 mg/kg daily × 4 doses; cyclosporine 2.5 mg/kg IV BID started		**1.0**	**0.0**	**32**	**130**
15		Perirectal abscess drained	**<0.1**	**0.0**	**32**	**127**
	Started filgrastim (5 µg/kg SC daily)		**0.2**	**0.0**	**34**	214
16	Filgrastim (5 µg/kg SC)		**0.6**	**0.0**	**33**	248
	Prednisone decreased to 30 mg once daily; filgrastim (5 µg/kg SC)		**1.2**	**0.0**	**35**	274
	Filgrastim (5 µg/kg SC)		6.1	**0.5**	**34**	276
	Filgrastim (5 µg/kg SC)		18.5	5.2	**34**	284
	Prednisone decreased to 20 mg daily		27.3	10.4	**35**	263
17	Prednisone decreased to 10 mg daily		19.6	12.5	37	158
	Prednisone decreased to 5 mg daily					
	Prednisone decreased to 5 mg every other day					
18			6.2	5.2	**32**	282
19			**3.4**	**1.6**	**37**	385
20	Prednisone dose increased back to 40 mg PO daily		**2.5**	**0.7**	**39**	252
21			**3.1**	2.3	40	267
	Prednisone decreased to 30 mg daily		7.3	4.9	41	239
	Prednisone 20 mg daily		8.8	6.6	43	241
24	Prednisone 10 mg daily		7.3	4.9	42	232
27	Prednisone 5 mg daily			7.9		
31	Prednisone 5 mg every other day			7.5		
34	Prednisone stopped			6.3		
36				5.4		

Abbreviations: WBC, white blood cell; ANC, absolute neutrophil count; Hct, hematocrit; UTI, urinary tract infection; PO, oral; BID, twice daily; IVIG, intravenous immunoglobulin; SC, subcutaneous.

a The values recorded in boldface fall outside of the normal range.

**Figure 2.**
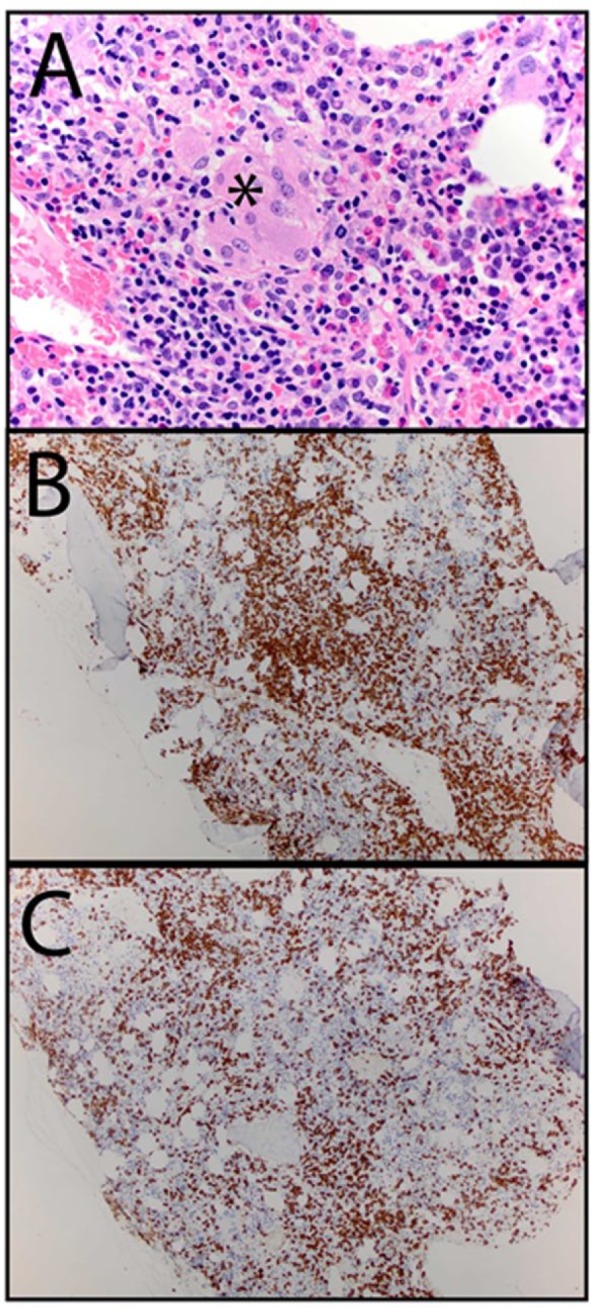
Bone marrow aspirate and biopsy12 weeks after the first ipilimumab infusion. The marrow revealed to be hypercellular with a prominent increase of bland histiocytes in the peritrabecular region (cluster marked with “*”; panel A; original magnification 400×). Well-formed (sarcoid-type) granulomas were not seen. In addition, there was lymphocytosis (bottom left, panel A). Most of the lymphocytes were CD8+ T cells (B: CD3; C: CD8), nonclonal, and with a negative T-cell receptor gene rearrangement test. Megakaryocytes were normal in number and morphology, and there was moderate eosinophilia. There was a striking and near complete absence of granulocyte precursors. A CD34 stain (a marker of the earliest neutrophil precursors) was positive in very rare cells. The differential count on the aspirate showed less than 2% mature and maturing granulocytes. The absence of myeloid precursors in the presence of a highly atypical immune infiltrate suggested that the neutropenia was due to an immune assault on the earliest myeloid forms.

A bone marrow aspirate and biopsy were obtained 12 weeks after the first ipilimumab infusion (Figure 2). The marrow was hypercellular with a prominent increase of bland histiocytes in the peritrabecular region (cluster marked with “*”; panel A; original magnification 400×). Well-formed (sarcoid-type) granulomas were not seen. In addition, there was lymphocytosis (bottom left, panel A). Most of the lymphocytes were CD8+ T cells (B: CD3; C: CD8), nonclonal, and with a negative T-cell receptor gene rearrangement test. Megakaryocytes were normal in number and morphology, and there was moderate eosinophilia. There was a striking and near complete absence of granulocyte precursors. A CD34 stain (a marker of the earliest neutrophil precursors) was positive in very rare cells. The differential count on the aspirate showed less than 2% mature and maturing granulocytes. The absence of myeloid precursors in the presence of a highly atypical immune infiltrate suggested that the neutropenia was due to an immune assault on the earliest myeloid forms.

## Diagnosis

Ipilimumab-induced neutropenia

## Management

No further ipilimumab was administered. Because of recurrence of fevers, he was admitted to the hospital on the 13th week after the first ipilimumab infusion. Since the leading differential diagnosis was immune-mediated agranulocytosis, he was started on prednisone 60 mg oral twice daily. Due to a lack of response in his granulocyte count after having been on prednisone for 6 days, he was started on cyclosporine 125 mg oral twice daily, immunoglobulin 40 gm IV daily for a total of 4 doses, and filgrastim 5 µg/kg subcutaneously daily for 5 days.

By day 9 of the cyclosporine/intravenous immunoglobulin/filgrastim regimen, the neutropenia still persisted with an ANC of 0·0 × 10^9^/L. His platelet count dropped slightly below normal (127 × 10^9^/L) by day 10 of this regimen, deemed likely secondary to antibiotics. Given the lack of improvement in the neutropenia, he was switched to a regimen of rabbit anti-thymocyte globulin (ATG) at 15 mg/kg IV daily for 4 doses and cyclosporine 2.5 mg/kg IV twice daily that was added to the prednisone at the end of week 14. This was patterned after a case report by Wei and colleagues,^1^ as detailed below in the discussion. Six days after starting the ATG/cyclosporine/prednisone regimen, his absolute monocyte count, which was 0.0 × 10^9^/L by 14 weeks subsequent to the first ipilimumab infusion, had increased to 0.1 × 10^9^/L. This prompted the reinstitution of filgrastim at 5 µg/kg SC daily. Nine days after starting the ATG/cyclosporine/prednisone regimen and 4 days after restarting filgrastim, his ANC increased from 0.0 × 10^9^/L the day prior to 0.5 × 10^9^/L, with normalization on the following day with an ANC of 5.2 × 10^9^/L. Of note, his ANC level started to recover approximately 7.5 weeks after his last dose of the ipilimumab.

By 19 weeks, the patient’s prednisone dose was tapered down to 5 mg every other day. At this juncture, his ANC dropped again to a nadir of 0.7 × 10^9^/L. Therefore, the prednisone dose was increased and a much slower taper was instituted over the course of approximately 4 additional months. Once the steroids were fully tapered off 5 months after the ATG treatment, the patient’s ANC finally stabilized to a normal level. His ANC remains normal now more than 6 months since the normalization of his ANC and 4 months since the prednisone was completely tapered off without any further intervention required.

## Conclusions

Cytotoxic T-lymphocyte antigen-4 (CTLA-4) is a cell surface molecule that is expressed nearly exclusively on CD4+ and CD8+ T cells. Studies have shown that the addition of anti-CTLA-4 monoclonal antibody leads to increased T-cell proliferation, presumably by blocking the interaction of CTLA-4 with its natural ligands CD80 and CD86.^2^ Ipilimumab is a human monoclonal IgG1 antibody against CTLA-4^3^ that is clinically used for the treatment of advanced melanoma, given studies that have demonstrated its ability to prolong survival.

The most common adverse events associated with ipilimumab are immune-related. These include enterocolitis, hepatitis, dermatitis, and hypophysitis. Severe hematological toxicity is rare. Enterocolitis is the most commonly reported adverse effect, occurring in 12.3% of patients; hypophysitis occurs as the second most frequent toxicity in 5% of the patients.^4,5^ CTLA-4 gene knock-out mice develop lymphoproliferative and autoimmune disorders.^6^

A direct effect of ipilimumab on neutrophils has not been described, as they lack CTLA-4 expression. In murine models, CTLA-4 is expressed on the surface of 11% to 15% of B cells, and blocking of CTLA-4 promotes B-cell effector function, thereby enhancing antibody production. It has also been shown that CTLA-4 is involved in downregulating interferon-γ release by CD8+ T cells. Experts postulate that such modes of immunomodulation may have myelosuppressive effects, such as the example seen with increased interferon levels in aplastic anemia. Thus, inactivation of CTLA-4 may be associated with enhanced antibody production.^7,8^

There are 2 other cases reported in the literature of neutropenia in patients receiving ipilimumab. In the first case, severe neutropenia occurred after the fourth treatment of ipilimumab (interestingly the same number of infusions as our case) and it rapidly reversed after intravenous immunoglobulin infusion, but did not respond to steroids.^9^ In the second case, there was severe neutropenia after ipilimumab infusion, but unlike our case, there was development of large granular lymphocytosis. This case responded rapidly to ATG, steroid, and cyclosporine therapy.^1^ In this case, the count recovery occurred approximately 7 weeks after the last infusion of the ipilimumab. The ANC of the patient we report recovered after administration of various immunosuppressive therapies. Clearance of ipilimumab, which has a half-life of 14.7 days, may have also contributed. It is intriguing that the recovery of ANC after ATG-based therapy in the report by Akhtari et al^9^ also occurred approximately 7 weeks after the last dose of the ipilimumab. There are no case reports of spontaneous recovery of severe neutropenia after ipilimumab, so the potential for recovery without intervention is unknown.

In summary, we report a case of severe neutropenia following ipilimumab therapy that fully resolved after the administration of prednisone, cyclosporine, and ATG. The temporal relationship between ipilimumab administration and the development of severe neutropenia, the absence of other inciting factors, and bone marrow biopsy findings consistent with immune-mediated suppression of myeloid progenitors implicate ipilimumab as the cause of neutropenia. Given the spectrum of autoimmune disorders associated with ipilimumab therapy and the potential for severe neutropenia as observed in this case and two others, we suggest that the complete blood count be monitored weekly in patients receiving ipilimumab. Furthermore, a prolonged steroid taper over the course of 4 to 6 months after the resolution of ipilimumab-induced neutropenia may be necessary to prevent a recurrence.

## References

[1] Wei G Nwakuche U Cadavid G Ajaz A Seiter K Liu D Large granular lymphocytosis with severe neutropenia following ipilimumab therapy for metastatic melanoma. Exp Hematol Oncol. 2012;1:3. doi:10.1186/2162-3619-1-3. 23210632 PMC3506991

[2] Linsley PS Greene JL Brady W Bajorath J Ledbetter JA Peach R Human B7-1 (CD80) and B7-2 (CD86) bind with similar avidities but distinct kinetics to CD28 and CTLA-4 receptors. Immunity. 1994;1:793-801. 7534620 10.1016/s1074-7613(94)80021-9

[3] Lee B Mukhi N Liu D Current management and novel agents for malignant melanoma. J Hematol Oncol. 2012;5:3. 22333219 10.1186/1756-8722-5-3 PMC3293076

[4] Beck KE Blansfield JA Tran KQ Enterocolitis in patients with cancer after antibody blockade of cytotoxic T-lymphocyte-associated antigen 4. J Clin Oncol. 2006;24:2283-2289. 16710025 10.1200/JCO.2005.04.5716 PMC2140223

[5] Blansfield JA Beck KE Tran K Cytotoxic T-lymphocyte-associated antigen-4 blockage can induce autoimmune hypophysitis in patients with metastatic melanoma and renal cancer. J Immunother. 2005;28:593-598. 16224277 10.1097/01.cji.0000178913.41256.06 PMC2154350

[6] Tivol EA Borriello F Schweitzer AN Lynch WP Bluestone JA Sharpe AH Loss of CTLA-4 leads to massive lymphoproliferation and fatal multiorgan tissue destruction, revealing a critical negative regulatory role of CTLA-4. Immunity. 1995;3:541-547. 7584144 10.1016/1074-7613(95)90125-6

[7] Quandt D Hoff H Rudolph M Fillatreau S Brunner-Weinzierl MC A new role of CTLA-4 on B cells in thymus-dependent immune responses in vivo. J Immunol. 2007;179:7316-7324. 18025174 10.4049/jimmunol.179.11.7316

[8] Pandiyan P Hegel JK Krueger M Quandt D Brunner-Weinzierl MC High IFN-gamma production of individual CD8 T lymphocytes is controlled by CD152 (CTLA-4). J Immunol. 2007;178:2132-4210. 17277117 10.4049/jimmunol.178.4.2132

[9] Akhtari M Waller EK Jaye DL Neutropenia in a patient treated with ipilimumab (anti-CTLA-4 antibody). J Immunother. 2009;32:322-324. 19242368 10.1097/CJI.0b013e31819aa40b

